# Development and validation of an interpretable model for predicting sepsis mortality across care settings

**DOI:** 10.1038/s41598-024-64463-0

**Published:** 2024-06-13

**Authors:** Young Seok Lee, Seungbong Han, Ye Eun Lee, Jaehwa Cho, Young Kyun Choi, Sun-Young Yoon, Dong Kyu Oh, Su Yeon Lee, Mi Hyeon Park, Chae-Man Lim, Jae Young Moon, the Korean Sepsis Alliance (KSA) Investigators, Sang‑Bum Hong, Suk‑Kyung Hong, Gee Young Suh, Kyeongman Jeon, Ryoung‑Eun Ko, Young‑Jae Cho, Yeon Joo Lee, Sung Yoon Lim, Sunghoon Park, Jeongwon Heo, Jae‑myeong Lee, Kyung Chan Kim, Youjin Chang, Sang‑Min Lee, Woo Hyun Cho, Sang Hyun Kwak, Heung Bum Lee, Jong‑Joon Ahn, Gil Myeong Seong, Song I. Lee, Tai Sun Park, Su Hwan Lee, Eun Young Choi, Hyung Koo Kang

**Affiliations:** 1 Division of Pulmonary, Allergy, and Critical Care Medicine, Department of Internal Medicine, Korea University Guro Hospital, Seoul, Republic of Korea; 2 Department of Biostatistics, Korea University College of Medicine, Seoul, Republic of Korea; 3 Division of Pulmonary and Critical Care Medicine, Department of Internal Medicine, Gangnam Severance Hospital, Yonsei University College of Medicine, Seoul, Republic of Korea; 4 Division of Infectious Disease and Critical Care Medicine, Department of Internal Medicine, Chungnam National University College of Medicine, Chungnam National University Sejong Hospital, Sejong, Republic of Korea; 5 Division of Pulmonary, Allergy, and Critical Care Medicine, Department of Internal Medicine, Chungnam National University College of Medicine, Chungnam National University Sejong Hospital, Sejong, Republic of Korea; 6 Department of Pulmonary and Critical Care Medicine, Asan Medical Center, University of Ulsan College of Medicine, Seoul, Republic of Korea; 7 Department of Critical Care Medicine, Samsung Medical Center, Sungkyunkwan University School of Medicine, Seoul, Republic of Korea; 8 Department of Pulmonary and Critical Care Medicine, Seoul National University Bundang Hospital, Seongnam, Republic of Korea; 9 Department of Pulmonary, Allergy and Critical Care Medicine, Hallym University Sacred Heart Hospital, Anyang, Republic of Korea; 10 Department of Internal Medicine, Kangwon National University Hospital, Chuncheon, Republic of Korea; 11 Department of Acute Care Surgery, Korea University Anam Hospital, Seoul, Republic of Korea; 12 Department of Internal Medicine, Daegu Catholic University Medical Center, Daegu Catholic University College of Medicine, Daegu, Republic of Korea; 13 Division of Pulmonary and Critical Care Medicine, Department of Internal Medicine, Inje University Sanggye Paik Hospital, Seoul, Republic of Korea; 14 Division of Pulmonary and Critical Care Medicine, Department of Internal Medicine, Seoul National University College of Medicine, Seoul, Republic of Korea; 15 Division of Allergy, Pulmonary and Critical Care Medicine, Department of Internal Medicine, Pusan National University Yangsan Hospital, Yangsan, Republic of Korea; 16 Department of Anesthesiology, Chonnam National University Hospital, Gwangju, Republic of Korea; 17 Department of Internal Medicine, Research Center for Pulmonary Disorders, Chonbuk National University Medical School, Jeonju, Republic of Korea; 18 Department of Pulmonary and Critical Care Medicine, Ulsan University Hospital, University of Ulsan College of Medicine, Ulsan, Republic of Korea; 19 Department of Internal Medicine, Jeju National University Hospital, Jeju National University School of Medicine, Jeju, Republic of Korea; 20 Division of Allergy, Pulmonary, and Critical Care Medicine, Department of Internal Medicine, Chungnam National University College of Medicine, Chungnam National University Hospital, Daejeon, Republic of Korea; 21 Department of Internal Medicine, Hanyang University College of Medicine, Seoul, Republic of Korea; 22 Division of Pulmonary and Critical Care Medicine, Department of Internal Medicine, Severance Hospital, Yonsei University College of Medicine, Seoul, Republic of Korea; 23 Department of Pulmonary and Allergy, Department of Internal Medicine, Regional Respiratory Center, Yeungnam University Hospital, Daegu, Republic of Korea; 24 Division of Pulmonary and Critical Care Medicine, Department of Internal Medicine, Inje University Ilsan Paik Hospital, Inje University College of Medicine, Goyang, Republic of Korea; 25 Division of Acute Care Surgery, Department of Surgery, Asan Medical Center, University of Ulsan College of Medicine, Seoul, Republic of Korea

**Keywords:** Sepsis, Mortality, Prognosis, Modeling, Point system, Diseases, Health care, Medical research, Risk factors

## Abstract

There are numerous prognostic predictive models for evaluating mortality risk, but current scoring models might not fully cater to sepsis patients’ needs. This study developed and validated a new model for sepsis patients that is suitable for any care setting and accurately forecasts 28-day mortality. The derivation dataset, gathered from 20 hospitals between September 2019 and December 2021, contrasted with the validation dataset, collected from 15 hospitals from January 2022 to December 2022. In this study, 7436 patients were classified as members of the derivation dataset, and 2284 patients were classified as members of the validation dataset. The point system model emerged as the optimal model among the tested predictive models for foreseeing sepsis mortality. For community-acquired sepsis, the model’s performance was satisfactory (derivation dataset AUC: 0.779, 95% CI 0.765–0.792; validation dataset AUC: 0.787, 95% CI 0.765–0.810). Similarly, for hospital-acquired sepsis, it performed well (derivation dataset AUC: 0.768, 95% CI 0.748–0.788; validation dataset AUC: 0.729, 95% CI 0.687–0.770). The calculator, accessible at https://avonlea76.shinyapps.io/shiny_app_up/, is user-friendly and compatible. The new predictive model of sepsis mortality is user-friendly and satisfactorily forecasts 28-day mortality. Its versatility lies in its applicability to all patients, encompassing both community-acquired and hospital-acquired sepsis.

## Introduction

Sepsis is a significant global health issue due to its steep mortality rates and economic impact. While our understanding and treatment of sepsis have evolved, sepsis-related deaths still account for an alarming 30–45% of global mortality, representing almost 20% of all deaths worldwide^1–4^. Considering that the prognosis of sepsis is influenced by an individual’s clinical condition and the nature of the pathogen, timely individual risk assessment using a prognostic predictive model is crucial. This allows for proper allocation of medical resources and can potentially reduce mortality^5–8^.

There are numerous prognostic predictive models for evaluating mortality risk^9–14^. Notably, the Acute Physiological and Chronic Health Assessment (APACHE) score and the Simple Acute Physiology Score (SAPS) are frequently employed in intensive care units (ICUs) to gauge mortality risk^10,11^. With evolving patient demographics in ICUs, such as increasing numbers of elderly, multimorbid, and immunocompromised patients, these scoring systems have been periodically updated to ensure effectiveness^10,15,16^. While beneficial, these models have limitations. Designed primarily for critically ill patients in ICUs, their application is typically upon ICU admission rather than at the initial diagnosis. Given that sepsis can be diagnosed in diverse settings, from ICUs to general wards or emergency rooms, and the crucial nature of timely intervention, current scoring models might not fully cater to sepsis patients’ needs. There is a pressing need for a predictive model that facilitates rapid mortality prediction and individualized treatment planning for sepsis patients across all care settings.

This study developed and validated a new model for sepsis patients that accurately predicts the 28-day mortality, which is user-friendly and suitable for any care setting. Particularly, we have prioritized ensuring that the final model is interpretable, thereby enhancing its usability for clinicians.

## Results

### Clinical characteristics of patients in the derivation dataset and the validation dataset

**Figure 1:**
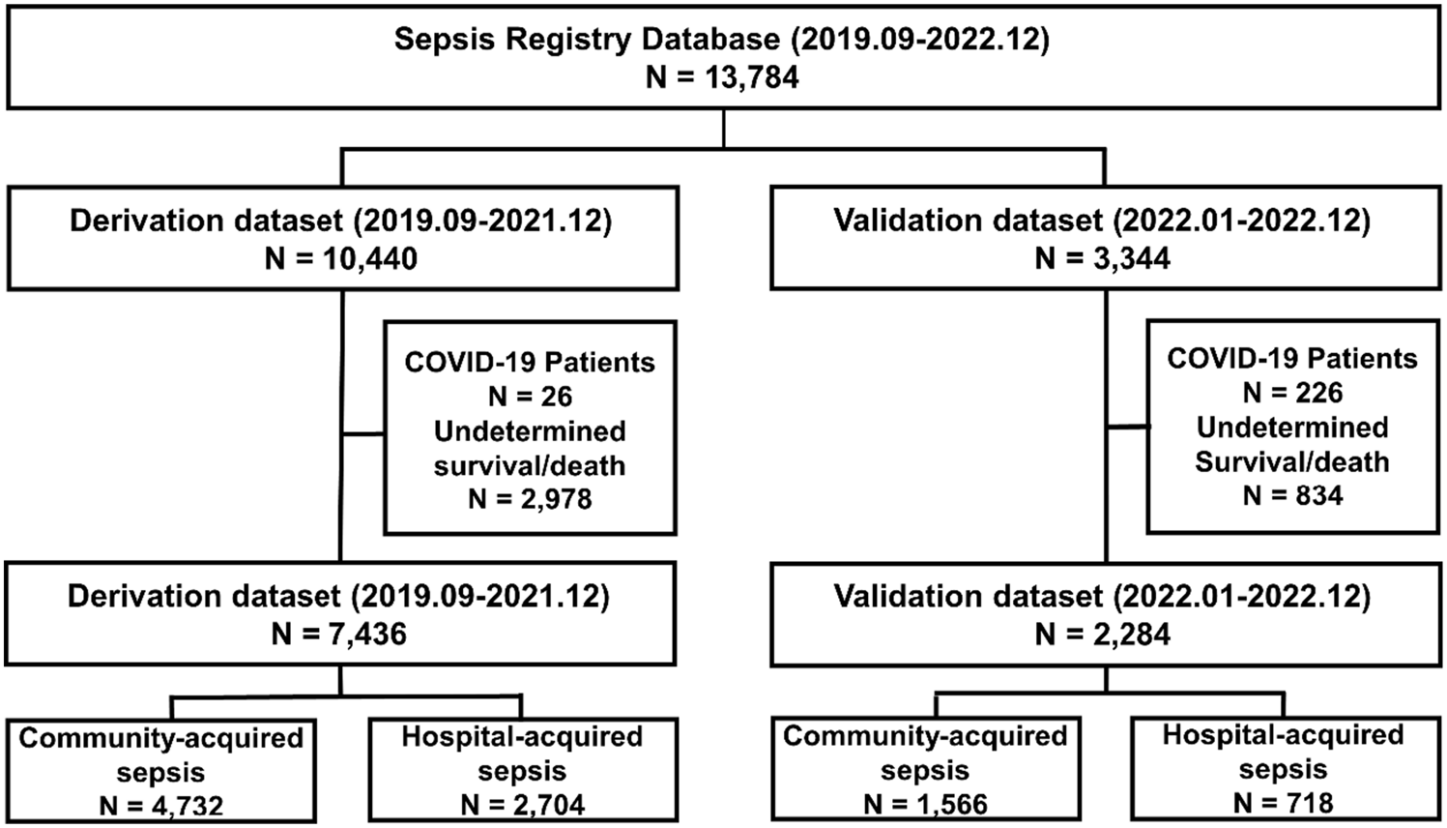
Flow chart of this study.

In the sepsis registry database, 10,440 patients were assigned to the derivation dataset, whereas 3344 patients were assigned to the validation dataset. Of these, 26 patients with coronavirus disease 2019 (COVID-19) in the derivation dataset and 226 patients with COVID-19 in the validation dataset were excluded. In addition, 2978 patients in the derivation dataset and 834 patients in validation dataset were excluded because of unclear survival status at 28 days after sepsis diagnosis. Finally, 7436 patients were included in the derivation dataset, and 2284 patients were included in the validation dataset (Fig. 1).

The clinical characteristics of the study population for the derivation and validation datasets are described in Table 1. In both datasets, nonsurvivors were older and more likely to be male compared to survivors. In addition, nonsurvivors had higher clinical frailty scale (CFS), sequential organ failure assessment (SOFA), and Charlson’s comorbidity index scores compared to survivors. Comorbidities were similar between the two groups, except malignancies. Sepsis caused by a respiratory infection had a poorer prognosis than sepsis caused by other infections. The use of steroids, ventilators, and continuous renal replacement therapy (CRRT) was higher in nonsurvivors than in survivors. Nonsurvivors had a lower blood pressure and body temperature and higher heart and respiratory rates compared to survivors at the time of diagnosis of sepsis. In addition, nonsurvivors had a greater rate of organ dysfunction based on initial laboratory findings compared to survivors at the time of diagnosis of sepsis (Supplementary Table S1).

**Table 1:** Clinical characteristics of study population in derivation dataset and validation dataset.

Variables	Derivation dataset			Validation dataset		
	Total (N = 7436)	Survivor (N = 4688)	Non-survivor (N = 2581)	Total (N = 2284)	Survivor (N = 1289)	Non-survivor (N = 995)
Age*	71 ± 14	70 ± 14	73 ± 13	72 ± 14	71 ± 14	73 ± 13
Male sex	4344 (58.4)	2687 (57.3)	1657 (60.3)	1314 (57.5)	728 (56.5)	586 (58.9)
Body mass index*	22 ± 4	22 ± 4	22 ± 4	23 ± 47	22 ± 4	24 ± 72
Clinical frailty scale*	5 ± 2	5 ± 2	6 ± 2	5 ± 2	5 ± 2	6 ± 2
CCI*	6 ± 3	5 ± 3	6 ± 3	6 ± 3	5 ± 3	6 ± 3
Comorbidities						
Cardiovascular disease	1323 (17.8)	797 (17)	526 (19.1)	484 (21.2)	263 (20.4)	221 (22.2)
Pulmonary disease	905 (12.2)	533 (11.4)	372 (13.5)	254 (11.1)	132 (10.2)	122 (12.3)
Neurologic disease	2343 (31.5)	1512 (32.3)	831 (30.2)	846 (37)	513 (39.8)	333 (33.5)
Liver disease	698 (9.4)	429 (9.2)	269 (9.8)	159 (7)	85 (6.6)	74 (7.4)
Diabetes mellitus	2481 (33.4)	1603 (34.2)	878 (32)	853 (37.3)	514 (39.9)	339 (34.1)
Chronic kidney disease	843 (11.3)	535 (11.4)	308 (11.2)	302 (13.2)	157 (12.2)	145 (14.6)
Connective tissue disease	192 (2.6)	131 (2.8)	61 (2.2)	69 (3)	41 (3.2)	28 (2.8)
Malignancy	3340 (44.9)	1879 (40.1)	1461 (53.2)	957 (41.9)	444 (34.4)	513 (51.6)
SOFA score, time zero*	7 ± 3	6 ± 3	8 ± 3	7 ± 3	6 ± 3	8 ± 3
Site of infection						
Respiratory infection	3269 (44)	1838 (39.2)	1431 (52.1)	1040 (45.5)	469 (36.4)	571 (57.4)
Gastrointestinal infection	2045 (27.5)	1403 (29.9)	642 (23.4)	510 (22.3)	327 (25.4)	183 (18.4)
Urinary tract infection	1051 (14.1)	831 (17.7)	220 (8)	354 (15.5)	278 (21.6)	76 (7.6)
Skin/soft tissue infection	189 (2.5)	132 (2.8)	57 (2.1)	79 (3.5)	52 (4)	27 (2.7)
Other	882 (11.9)	484 (10.4)	398 (14.4)	301 (13.2)	163 (12.6)	138 (13.9)
Type of infection						
Community-acquired	4732 (63.6)	2991 (63.8)	1741 (63.4)	1566 (68.6)	905 (70.2)	661 (66.4)
Hospital-acquired	2704 (36.4)	1697 (36.2)	1007 (36.6)	718 (31.4)	384 (29.8)	334 (33.6)
Steroid use	1080 (14.5)	593 (12.6)	487 (17.7)	386 (16.9)	201 (15.6)	185 (18.6)
ICU admission	3612 (48.6)	2357 (50.3)	1255 (45.7)	949 (41.5)	603 (46.8)	346 (34.8)
Ventilator use	2023 (27.2)	1097 (23.4)	926 (33.7)	542 (23.7)	276 (21.4)	266 (26.7)
CRRT use	1081 (14.5)	451 (9.6)	630 (22.9)	292 (12.8)	124 (9.6)	168 (16.9)
Hospital LOS (days)*	26 ± 64	34 ± 76	11 ± 16	22 ± 28	29 ± 31	11 ± 19

*CCI* Charlson comorbidity index, *SOFA* sequential organ failure assessment, *ICU* intensive care unit, *CRRT* continuous renal replacement therapy, *LOS* length of stay.

*Data are presented as means ± standard deviations. Other variables are presented as numbers and percentages.

### New models for predicting 28-day mortality in sepsis patients

In the derivation dataset, multivariable logistic regression identified the following as significant predictors for 28-day mortality: age, CFS, presence of malignancy, SOFA score, sepsis originating from respiratory infections, use of CRRT, body temperature, albumin levels, international normalized ratio (INR), C-reactive protein (CRP) levels, and lactic acid levels at the time of sepsis diagnosis (Supplementary Table S2). These 11 factors were used as predictive variables across the seven prediction models.

**Figure 2:**
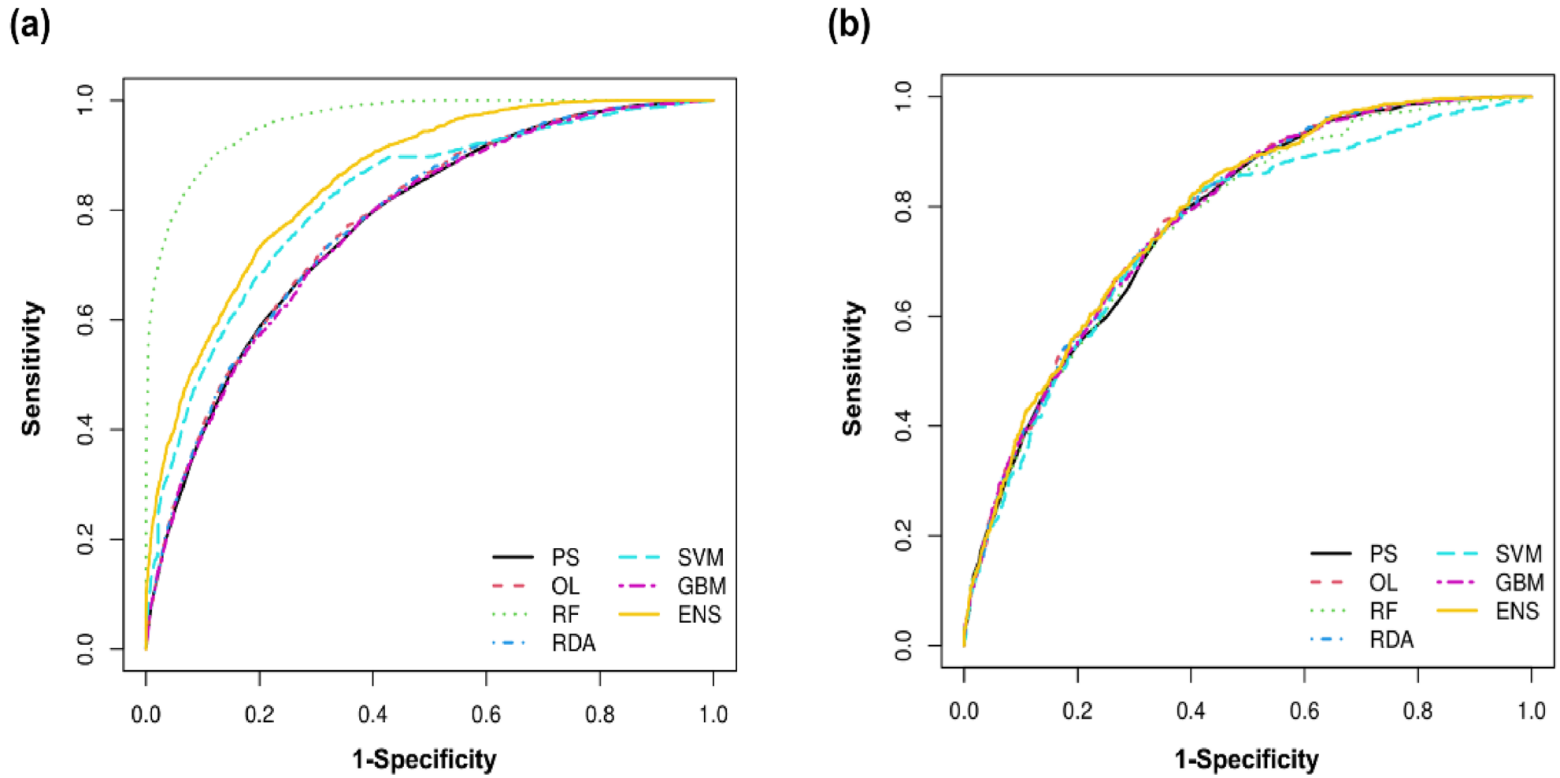
Comparison of 28-day mortality predictive ability among point system (PS), ensemble method (ENS), ordinary logistic regression (OL), regularized discriminant analysis (RDA), random forest (RF), support vector machine (SVM), and gradient-boosting machine (GBM). (**a**) Derivation dataset. (**b**) Validation dataset.

To identify the most precise models for predicting sepsis mortality, we assessed the point system (PS), ordinary logistic regression (OL), random forest (RF), regularized discriminant analysis (RDA), support vector machine (SVM), gradient-boosting machine (GBM), and ensemble method (ENS) predictive models using multiple metrics. Based on the analysis of the area under the receiver operating characteristic curve (AUC), calibration plots, the Hosmer–Lemeshow test statistic, and Brier score in both datasets, including cross-validation, the performance of the PS model demonstrated similarity to that of other models in predicting sepsis mortality (Fig. 2 and Supplementary Tables S3–5). Additionally, the PS model’s ease of interpretation for clinical application led to its selection as the final model.

The final predictive model for sepsis mortality was structured as follows: Age (scored from 0 for < 40 years to 7 for > 80 years in increments based on decade ranges), CFS (scored from 0 for 1–3, up to 7 for 6–9), presence of malignancy (7 points), SOFA score (scored from 0 for 2–7 up to 13 for 18–21), sepsis due to respiratory infection (6 points), CRRT usage (7 points), body temperature (scored from 0 for > 38℃ to 7 for < 36 ℃), albumin level (scored from 0 for ≥ 3.5 g/dL to 7 for < 2.5 g/dL), INR (> 1.3 earns 3 points), CRP level (scored from 0 for ≤ 10 mg/dL up to 2 for > 20 mg/dL), and lactic acid level (scored as 0 for < 4 mmol/L and 7 for ≥ 4 mmol/L). The maximum attainable score was 73 points (Table 2). The associated mortality risk based on the total score can be found in Supplementary Table S6.

**Table 2:** New predictive model of sepsis mortality.

Variables	Categories	Scores
Age	< 40 years	0
	40–49 years	2
	50–59 years	3
	60–69 years	5
	70–79 years	6
	≥ 80 years	7
Clinical frailty scale	1–3 points	0
	4–5 points	4
	6–9 points	7
Presence of malignancy	Yes	7
SOFA score	2–7 points	0
	8–12 points	5
	13–17 points	9
	18–21 points	13
Respiratory infection	Yes	6
CRRT use	Yes	7
Body temperature	> 38 ℃	0
	36 ℃ ≤ body temperature ≤ 38 ℃	4
	< 36 ℃	7
Albumin	≥ 3.5 g/dL	0
	2.5 g/dL ≤ albumin < 3.5 g/dL	4
	< 2.5 g/dL	7
INR	> 1.3	3
CRP	≤ 10 mg/dL	0
	10 mg/dL < CRP ≤ 20 mg/dL	1
	> 20 mg/dL	2
Lactic acid	< 4 mmol/L	0
	≥ 4 mmol/L	7
Total score		73

*SOFA* sequential organ failure assessment, *CRRT* continuous renal replacement therapy, *INR* international normalized ratio, *CRP* C-reactive protein.

### New predictive model of sepsis mortality in different clinical situations

**Figure 3:**
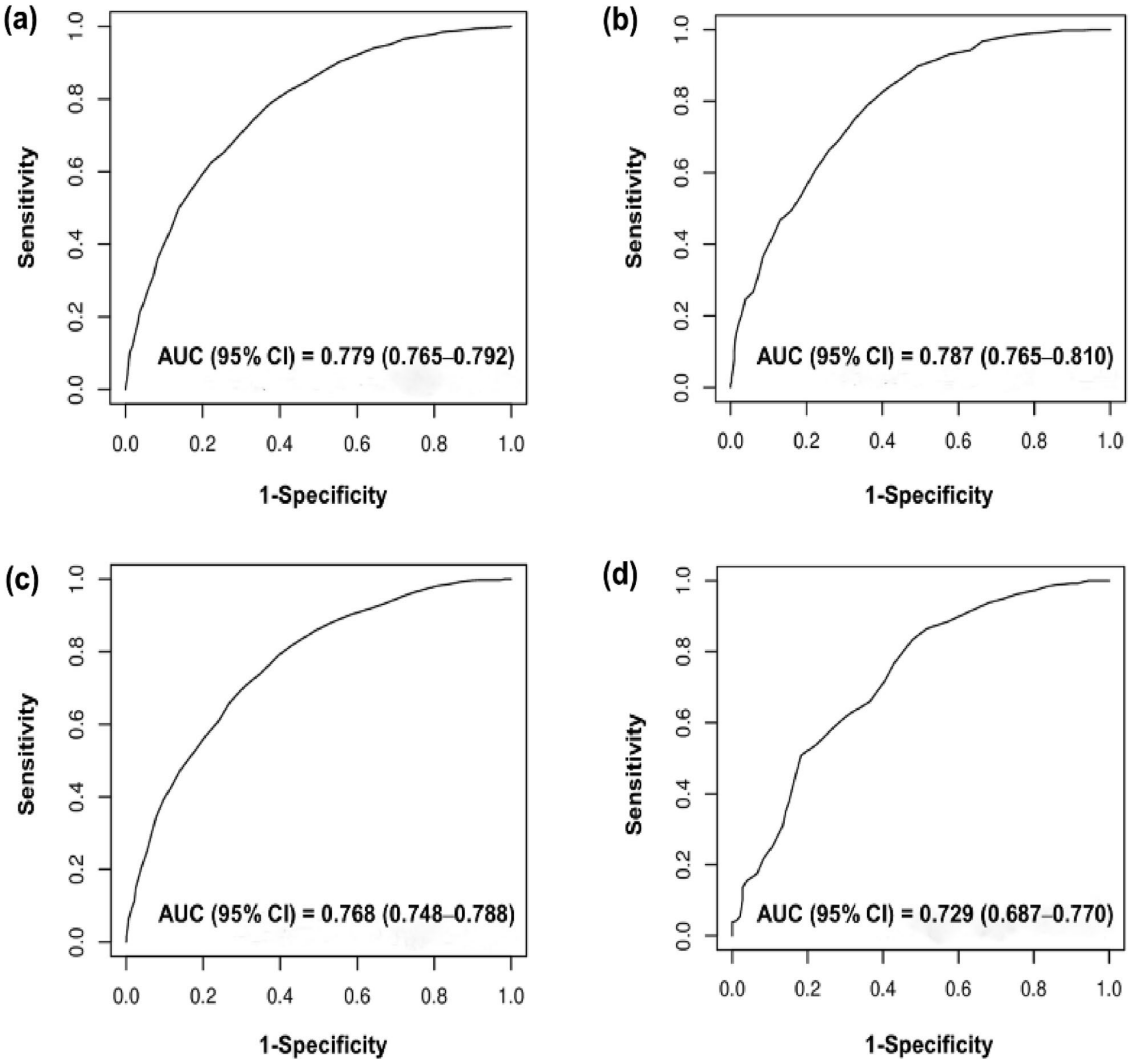
Performance of the new predictive model for predicting sepsis mortality in community-acquired sepsis and hospital-acquired sepsis. The 4 panels show receiver operating characteristic curves for patients in the (**a**) derivation dataset in community-acquired sepsis, (**b**) validation dataset in community-acquired sepsis, (**c**) derivation dataset in hospital-acquired sepsis, and (**d**) validation dataset in hospital-acquired sepsis after predicting 28-day mortality according to their score using the new predictive model.

To assess the efficacy of the new model across diverse clinical scenarios, we examined the ROC curve in both the derivation and validation datasets for community-acquired and hospital-acquired sepsis. For community-acquired sepsis, the model’s performance was satisfactory (derivation dataset AUC: 0.779, 95% CI 0.765–0.792; validation dataset AUC: 0.787, 95% CI 0.765–0.810; Fig. 3). Similarly, for hospital-acquired sepsis, it performed well (derivation dataset AUC: 0.768, 95% CI 0.748–0.788; validation dataset AUC: 0.729, 95% CI 0.687–0.770; Fig. 3).

**Figure 4:**
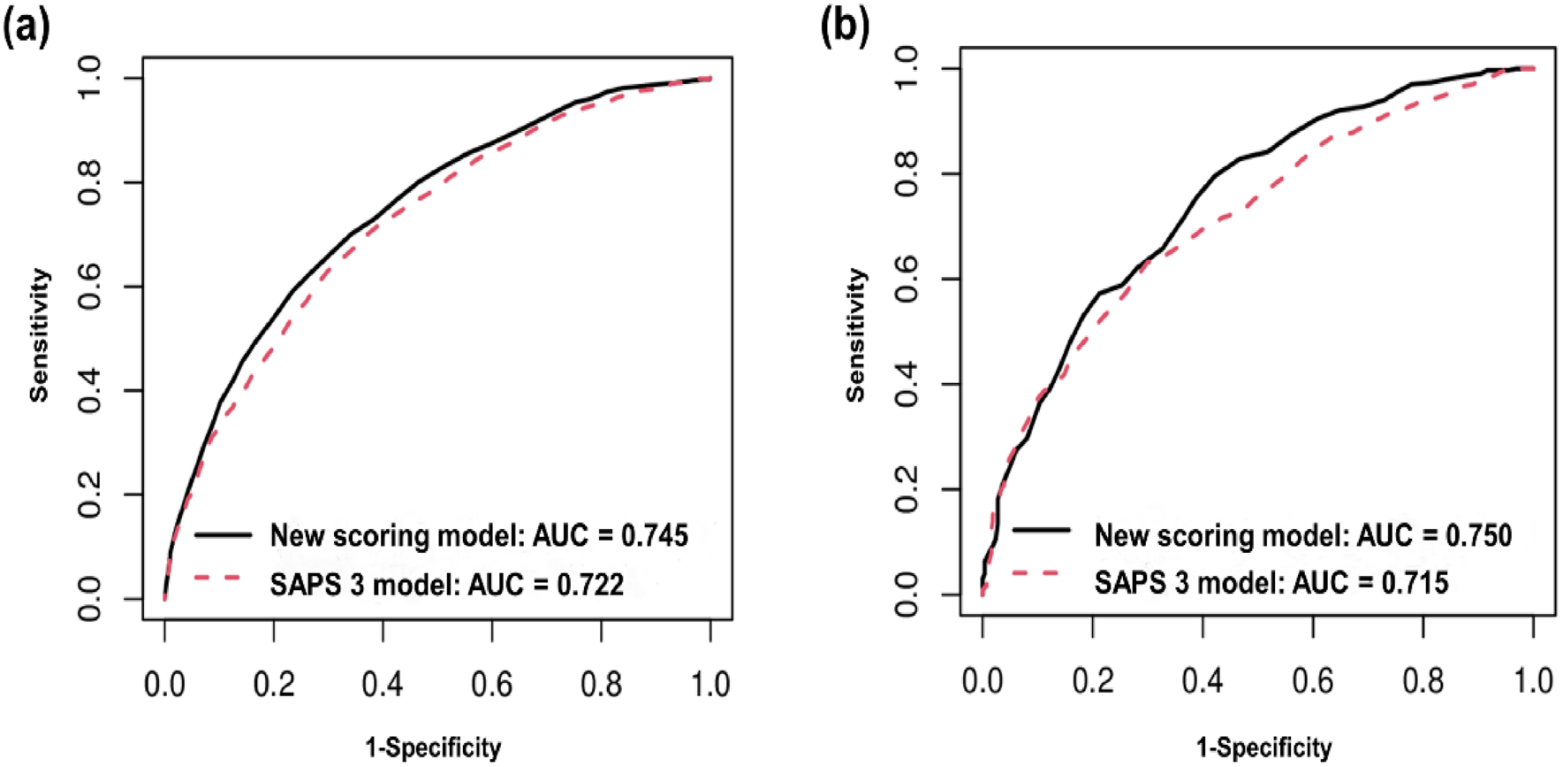
Comparison of 28-day mortality predictive ability between the new predictive model and simplified acute physiology score III(SAPS 3) in sepsis patients admitted to an intensive care unit. The new predictive model of sepsis mortality was more accurate than the SAPS 3 scoring system in predicting 28-day mortality from sepsis in critically ill patients. (**a**) Derivation dataset (new predictive model vs. SAPS 3, 0.745 vs. 0.722; difference in AUC; 95% CI 0.005–0.042; P = 0.012). (**b**) Validation dataset (new predictive model vs. SAPS 3, 0.750 vs.0.715; difference in AUC; 95% CI − 0.001–0.071; P = 0.063.

To further assess its efficacy for predicting outcomes for critically ill patients, we compared its ability to predict 28-day mortality against the established SAPS 3 system using both datasets. Among the 3,612 critically ill sepsis patients in the derivation dataset, the performance of the new scoring model (AUC: 0.745) was comparable to that of the SAPS 3 model (AUC: 0.722) (difference in AUC; 95% CI 0.005–0.042; P = 0.012), indicating similar predictive accuracy. Similarly, in the validation dataset comprising 949 critically ill sepsis patients, the new model (AUC: 0.750) tended to show statistically insignificant non-inferior predictive accuracy compared to SAPS (difference in AUC; 95% CI − 0.001 to 0.071; P = 0.063) (Fig. 4).

### Clinical utility of new predictive model of sepsis mortality

To enhance the clinical utility of the new model, we developed a calculator app using shinyapps.io. The calculator, accessible at https://avonlea76.shinyapps.io/shiny_app_up/, is user-friendly and compatible with both smartphones and computers (e.g. electronic medical record). Given the criticality of swift decision-making for patients with sepsis, this app promises to be an invaluable tool for predicting sepsis mortality in clinical settings.

## Discussion

This study introduced a new model for predicting 28-day mortality among sepsis patients, incorporating 11 variables. The model exhibited commendable performance across various clinical scenarios, including both community- and hospital-acquired sepsis, positioning it as a valuable instrument for assessing mortality risk across all sepsis patients.

Despite advancements in sepsis strategies informed by numerous studies, the overall prognosis for sepsis remains suboptimal^1–4^. For better outcomes, it is imperative for clinicians to differentiate between patients with likely favorable outcomes and those at higher risk, ensuring tailored treatment approaches. Mismanagement of medical resources can escalate mortality rates, which makes efficient resource allocation vital^17–19^. If prognostic predictions can be made at the time of sepsis diagnosis, those with a poorer outlook could be prioritized for ICU beds and resources over those with a more optimistic prognosis.

While established scoring models such as APACHE and SAPS are renowned for their predictive accuracy across diverse cohorts of critically ill patients^14^, they primarily target this patient subset and not the broader population. Their adaptation for sepsis patients might be less than ideal. Following the revised sepsis-3 definition^20^, new prognostic models (encompassing biomarker models, immune dysfunction scores, and machine-learning models) have been developed and validated^13,21–26^. Yet, small sample sizes and restricted accessibility limit some, such as the biomarker model and immune dysfunction score^25,26^. Machine-learning models, although superior in terms of predictive accuracy compared to older scoring models, are typically institution-specific and their performance may not be generalizable. Notably, any lack of laboratory data can postpone the presentation of results^13,21,22^.

The sepsis mortality prediction model presented here has noteworthy advantages over its predecessors. First, this study established an optimal scoring point system for predicting the sepsis mortality among seven predictive models. The predictive models ranged from original logistic regression models to the latest machine learning models.

Based on the AUC, calibration plots, Hosmer–Lemeshow test statistics, and Brier score, the PS model did not exhibit inferior performance to other machine learning techniques in the fivefold cross validation and the validation dataset. In fact, it demonstrated superior performance in terms of calibration. In addition, the PS model offered interpretive advantages, leading to its selection as the final prediction model. We concluded that our model is the most suitable among several models for predicting sepsis mortality. Second, its applicability spans both community- and hospital-acquired sepsis, demonstrating robust performance in both patient categories (Fig. 3). Furthermore, it consistently demonstrated performance comparable to SAPS in terms of predicting outcomes, even among critically ill sepsis patients (Fig. 4). Third, the model’s design readily accommodates real-time clinical data, enabling prompt prognosis predictions concurrent with sepsis diagnosis. Fourth, to augment the practicality of our model, we developed a user-friendly calculator app, which provides rapid mortality prediction, facilitating informed decision-making regarding clinical management and potentially mitigating sepsis-associated mortality rates. Finally, the model encompasses foundational clinical parameters such as age, CFS, and malignancy presence, infection sites, inflammation metrics, and severity indicators (including organ dysfunction) making it clinically intuitive. Notably, the inclusion of the CFS, recently identified as a predictor of sepsis prognosis, strengthens the model^27^.

However, this study had several limitations. First, potential selection bias arose in determining survival outcomes for patients discharged within 28 days after sepsis diagnosis. Second, only Korean patients participated in this study. However, the number of included patients was relatively large. Finally, the sepsis mortality rate in this study was relatively high because it was conducted in a university-affiliated hospital. During the study period, these hospitals admitted more severe patients compared to other periods, primarily because of the low medical resources during the COVID-19 pandemic. Despite these limitations, our model was developed using a relatively large sample size and diverse modeling techniques. In addition, the calculator based on our model is user-friendly and convenient for clinical settings.

In conclusion, the new predictive model of sepsis mortality is user-friendly and effectively forecasts 28-day mortality. Its versatility lies in its applicability to all patients, encompassing both community-acquired and hospital-acquired sepsis, at the time of diagnosis. To further validate this novel model, multinational studies with larger cohorts across diverse scenarios are needed.

## Methods

### Nationwide multicenter prospective sepsis cohort

The cohort was supported by a research program from the Korea Disease Control and Prevention Agency. Clinical data were amassed by the Korean Sepsis Alliance (KSA), encompassing tertiary referral and university-affiliated hospitals, to study the epidemiology, clinical practices, and outcomes of sepsis patients in the Republic of Korea. Between September 2019 and December 2021, 20 hospitals contributed to the sepsis cohort project (KSA 3 database). From January 2022 to December 2022, this participation narrowed to 15 hospitals (KSA 4 database). The database differentiated patients into community-acquired sepsis (diagnosed in emergency rooms) and hospital-acquired sepsis (diagnosed 48 h post-admission to a general ward). The study cohort comprised adults aged ≥ 19 years diagnosed with life-threatening organ dysfunction caused by a dysregulated host response to infection, characterized by a ≥ 2-point increase in the SOFA score^20^. Data spanned from “time zero” (either when community-acquired sepsis was identified in emergency rooms or when hospital-acquired sepsis was diagnosed by medical professionals after 48 h of admission in the general ward) until hospital discharge or death^27–31^.

### Study design

This research was a multicenter prospective observational cohort study using data from the Korean Sepsis Alliance’s sepsis cohort. The derivation dataset (KSA 3) was gathered from 20 hospitals between September 2019 and December 2021; the validation dataset (KSA 4) was collected from 15 hospitals between January 2022 and December 2022. We designed and assessed various predictive models for sepsis mortality using both datasets, aiming to select the most accurate model. Subsequently, we developed an app to implement the final model, facilitating its clinical application. The primary outcome was the 28-day mortality post-sepsis diagnosis. Due to the lack of follow-up data in the KSA database, we established an arbitrary definition for 28-day mortality following the diagnosis of sepsis. For patients discharged more than 28 days after the diagnosis of sepsis, the 28-day mortality was recorded. However, patients discharged within 28 days were categorized as survivors if they received antibiotics for more than 7 days and did not require life-sustaining treatment. This decision was based on the observation that most infections are treated with antibiotics for more than 7 days after the diagnosis. Patients from other categories were excluded due to uncertainty regarding their 28-day mortality status. In addition, patients with coronavirus disease 2019 (COVID-19) were excluded due to the potential impact on clinical outcomes.

### Definition of variables

The CFS is a robustly validated nine-point scale that classifies patients based on clinical insight. It was used at sepsis diagnosis, leveraging clinical data from up to 2 weeks prior to diagnosis^27,32,33^. Comorbid patients had prior disease diagnoses at the sepsis detection time, and the infection site was identified as the infection’s origin. Comorbidities and infection sites were classified following the management of severe sepsis in Asia’s intensive care units (MOSAICS) study method^2,34^. Malignancy, including hematological and solid tumors, indicated current malignancy presence at sepsis diagnosis. Vital signs and laboratory results were recorded at sepsis diagnosis; the usages of ventilators and CRRT were determined based on need at sepsis diagnosis.

### Statistical analysis

We followed the Transparent Reporting of a Multivariable Prediction Model for Individual Prognosis or Diagnosis (TRIPOD) guidelines in developing and validating a 28-day mortality predictive model for sepsis patients^35^. All statistical analyses were conducted using R software (http://www.r-project.org). Descriptive statistics for both derivation and validation datasets are presented as means ± standard deviation (SDs) or frequencies with percentages for continuous and categorical variables, respectively. For predicting 28-day mortality, various models including OL, RDA, and three machine learning algorithms (RF, SVM, and GBM) were employed.

The RDA employs a classification rule rooted in regularized group covariance matrices, targeting enhancement against multicollinearity of covariates. The RF, an ensemble learning theory derivative, produces multiple decision trees during training^36^. The final class prediction arises from the majority prediction across all trees. RF effectively captures both simple and intricate classification functions by recognizing predictor interactions. SVM identifies a hyperplane in a high-dimensional space that distinctly separates data points of varying classes. It showcases robustness in processing high-dimensional data and employs an influential regularization method to prevent overfitting^37^. GBM, another ensemble learning algorithm, strengthens predictions by progressively optimizing the log-likelihood loss function, starting from a base model^38^. Its strengths include discerning complex nonlinear relationships and adeptly managing diverse data types. In addition, we contemplated two other techniques for final model selection: the PS^39^ and an ENS amalgamating OL, RF, RDA, SVM, and GBM, which averaged mortality probabilities for a final prediction. Among machine learning models, various options exist for hyperparameter selection, including grid search, random search, genetic algorithm, and Bayesian optimization. However, current research suggests that none of these methods are extensively employed. Typically, the default option is widely utilized in medical data modeling^40–42^. Nonetheless, hyperparameter selection was conducted for RF. The hyperparameters were determined through five-fold cross-validation on the derivation dataset; the R package tuneRanger was employed for hyperparameter selection during RF modeling. The hyperparameters for other machine learning techniques were selected based on the default settings.

To pinpoint pivotal predictive variables, univariable logistic regression models were initially fitted, with variables with a p-value < 0.1 becoming primary candidates for predictive models. Multicollinearity was addressed by iterative removal of the least significant variable in the multivariable logistic regression model, scrutinized via the variation inflation factor index (VIF). Following intensive consultations with clinical and statistical experts, we chose 11 predictive variables for the concluding model. For continuous variables such as age, CRP level, and SOFA score, which were included in the logistic model, linearity was examined using multivariable fractional polynomial models based on the R package *mfp *(Multi-variable Fractional Polynomials)^43^. Considering that no significant nonlinear relationships were observed, the logistic model was fitted in its original scale without any variable transformations. We also fitted a PS model based on a logistic model. Unlike other machine learning models, a risk score was assigned to each risk factor, which has interpretive advantages. For this purpose, continuous variables were categorized and scored according to the level of each category corresponding to a patient profile. Each patient’s risk characteristics were assigned a score, which was used to determine the overall risk. PS models provide interpretable results compared with black box models, thus facilitating decision-making for clinical researchers involved in patient care. However, PS models require an underlying base model, and thus, the logistic model previously fitted is considered the base model. The main principle comprises approximating the linear combination of covariates as an overall risk score with respect to the risk. For detailed construction of the PS model, please refer to Sullivan et al., Zhang et al., and Greving et al.^39,44,45^. In the derivation dataset, the rate of missing data was not high. Variables with a relatively high rate of missing data were body mass index (4.78%), international normalized ratio (INR) (4.96%), and albumin (1.29%), all of which were < 5%. Indeed, 6730 individuals from the derivation dataset and 2033 individuals from the validation dataset, after excluding those with significant missing data, were included in the analyses for model fitting. We performed model fitting by categorizing variables with well-established categories. In general, ML methods do not require variable categorization. However, the creation of a PS model requires categorization. In this case, we categorized variables according to clinical criteria or socially recognized categories (e.g. age). The categories for continuous variables were decided based on the ease of model interpretability. For example, CFS and body temperature values were derived from prior studies^27,30^. Values for albumin, INR, and lactate were established based on clinical significance (for instance, albumin levels: ≥ 3.5 g/dL as normal, 2.5 g/dL ≤ albumin < 3.5 g/dL as low, and < 2.5 g/dL indicating significant hypoalbuminemia; INR > 1.3 as abnormal; lactic acid ≥ 4 mmol/L as hyperlactatemia). Furthermore, no scaling or other variable transformations for continuous variables were performed prior to PS and logistic regression modeling. However, in other machine learning techniques, continuous variables were standardized before modeling.

Model performances of PS, OL, RF, RDA, SVM, GBM, and ENS were gauged through various metrics: the area under the AUC, calibration plots, the Hosmer–Lemeshow test statistic, and the Brier score. The AUC gauges patient risk discrimination, and the calibration plot juxtaposes predicted against observed probabilities. For calibration assessment, patients were categorized into 10 risk factions. Recognizing the known calibration measurement inefficiencies of the Hosmer–Lemeshow test, we abstained from p-value calculations, emphasizing test statistic model comparisons instead.

Internal validation was conducted to evaluate the apparent performance of the derivation set using the AUC, Hosmer–Lemeshow test statistics, and Brier score. Additionally, fivefold cross-validation was employed to assess predictive performance. Furthermore, external validation was conducted to evaluate model performance in the validation dataset. In addition, our final model was juxtaposed with the SAPS 3 scoring system using Delong’s AUC comparison method^46^. RF, RDA, SVM, and GBM implementations employed R packages: randomForest, klaR, e1071, and mboost, respectively^47–50^. ROC curves were used to gauge the discrimination capability of the predictive model across clinical scenarios such as community-acquired or hospital-acquired sepsis, evaluated via R packages pROC and ‘predictABEL’. All p-values were two-sided, with values under 0.05 deemed statistically significant.

### Ethics statement

The study received approval from the institutional review board (IRB) of each participating hospital (Supplementary Tables S7, 8). In alignment with the principles outlined in the Declaration of Helsinki, we prioritized patient privacy and confidentiality. The requirement for written informed consent was waived by the IRBs of the participating hospitals.

### Supplementary Information

Supplementary Tables.

## Data Availability

Data will be shared upon reasonable request to the corresponding author only after permission by the institutional review board of each participating hospital.

## References

[1] Rudd KE (2020). Global, regional, and national sepsis incidence and mortality, 1990–2017: Analysis for the Global Burden of Disease Study. Lancet (Lond., Engl.).

[2] Li A (2022). Epidemiology, management, and outcomes of sepsis in ICUs among countries of differing national wealth across Asia. Am. J. Respir. Crit. Care Med..

[3] Markwart R (2020). Epidemiology and burden of sepsis acquired in hospitals and intensive care units: A systematic review and meta-analysis. Intensive Care Med..

[4] Bauer M (2020). Mortality in sepsis and septic shock in Europe, North America and Australia between 2009 and 2019- results from a systematic review and meta-analysis. Crit. Care (Lond., Engl.).

[5] McGrath SP, MacKenzie T, Perreard I, Blike G (2021). Characterizing rescue performance in a tertiary care medical center: A systems approach to provide management decision support. BMC Health Serv. Res..

[6] Zhang Z, Ho KM, Gu H, Hong Y, Yu Y (2020). Defining persistent critical illness based on growth trajectories in patients with sepsis. Crit. Care (Lond., Engl.).

[7] Gavelli F, Castello LM, Avanzi GC (2021). Management of sepsis and septic shock in the emergency department. Intern. Emerg. Med..

[8] Evans L (2021). Surviving sepsis campaign: International guidelines for management of sepsis and septic shock 2021. Intensive Care Med..

[9] Quintairos A, Pilcher D, Salluh JIF (2022). ICU scoring systems. Intensive Care Med..

[10] Zimmerman JE, Kramer AA, McNair DS, Malila FM (2006). Acute Physiology and Chronic Health Evaluation (APACHE) IV: Hospital mortality assessment for today's critically ill patients. Crit. Care Med..

[11] Le Gall JR, Lemeshow S, Saulnier F (1993). A new simplified acute physiology score (SAPS II) based on a European/North American multicenter study. JAMA.

[12] Lemeshow S (1993). Mortality probability models (MPM II) based on an international cohort of intensive care unit patients. JAMA.

[13] Goh KH (2021). Artificial intelligence in sepsis early prediction and diagnosis using unstructured data in healthcare. Nat. Commun..

[14] Haniffa R, Isaam I, De Silva AP, Dondorp AM, De Keizer NF (2018). Performance of critical care prognostic scoring systems in low and middle-income countries: a systematic review. Crit. Care (Lond., Engl.).

[15] Flaatten H (2017). The status of intensive care medicine research and a future agenda for very old patients in the ICU. Intensive Care Med..

[16] Nassar AP, Malbouisson LM, Moreno R (2014). Evaluation of simplified acute physiology score 3 performance: A systematic review of external validation studies. Crit. Care (Lond., Engl.).

[17] Emanuel EJ (2020). Fair allocation of scarce medical resources in the time of Covid-19. N. Engl. J. Med..

[18] Sabatello M, Burke TB, McDonald KE, Appelbaum PS (2020). Disability, ethics, and health care in the COVID-19 pandemic. Am. J. Public Health.

[19] Kirkpatrick JN, Hull SC, Fedson S, Mullen B, Goodlin SJ (2020). Scarce-resource allocation and patient triage during the COVID-19 pandemic: JACC review topic of the week. J. Am. Coll. Cardiol..

[20] Singer M (2016). The third international consensus definitions for sepsis and septic shock (sepsis-3). JAMA.

[21] Kong G, Lin K, Hu Y (2020). Using machine learning methods to predict in-hospital mortality of sepsis patients in the ICU. BMC Med. Inform. Decis. Mak..

[22] Li K, Shi Q, Liu S, Xie Y, Liu J (2021). Predicting in-hospital mortality in ICU patients with sepsis using gradient boosting decision tree. Medicine.

[23] Khwannimit B, Bhurayanontachai R, Vattanavanit V (2017). Validation of the sepsis severity score compared with updated severity scores in predicting hospital mortality in sepsis patients. Shock (Augusta, Ga).

[24] Hou N (2020). Predicting 30-days mortality for MIMIC-III patients with sepsis-3: A machine learning approach using XGboost. J. Transl. Med..

[25] Fang WF (2017). Development and validation of immune dysfunction score to predict 28-day mortality of sepsis patients. PLoS One.

[26] Mikacenic C (2017). A two-biomarker model predicts mortality in the critically ill with sepsis. Am. J. Respir. Crit. Care Med..

[27] Lee HY (2022). Preexisting clinical frailty is associated with worse clinical outcomes in patients with sepsis. Crit. Care Med..

[28] Hyun DG (2022). Mortality of patients with hospital-onset sepsis in hospitals with all-day and non-all-day rapid response teams: A prospective nationwide multicenter cohort study. Crit. Care (Lond., Engl.).

[29] Im Y (2022). Time-to-antibiotics and clinical outcomes in patients with sepsis and septic shock: A prospective nationwide multicenter cohort study. Crit. Care (Lond., Engl.).

[30] Park S (2020). Normothermia in patients with sepsis who present to emergency departments is associated with low compliance with sepsis bundles and increased in-hospital mortality rate. Crit. Care Med..

[31] Jeon K (2019). Characteristics, management and clinical outcomes of patients with sepsis: A multicenter cohort study in Korea. Acute Crit. Care.

[32] Rockwood K (2005). A global clinical measure of fitness and frailty in elderly people. CMAJ Can. Med. Assoc. J. Assoc. Med. Can..

[33] Moorhouse P, Rockwood K (2012). Frailty and its quantitative clinical evaluation. J. R. Coll. Physicians Edinb..

[34] Phua J (2011). Management of severe sepsis in patients admitted to Asian intensive care units: Prospective cohort study. BMJ (Clin. Res. Ed.).

[35] Moons KG (2015). Transparent reporting of a multivariable prediction model for individual prognosis or diagnosis (TRIPOD): Explanation and elaboration. Ann. Intern. Med..

[36] Breiman LJML (2001). Random forests. Mach. Learn..

[37] Statnikov A, Wang L, Aliferis CF (2008). A comprehensive comparison of random forests and support vector machines for microarray-based cancer classification. BMC Bioinform..

[38] Natekin A, Knoll A (2013). Gradient boosting machines, a tutorial. Front. Neurorobot..

[39] Sullivan LM, Massaro JM, D'Agostino RB (2004). Presentation of multivariate data for clinical use: The Framingham Study risk score functions. Stat. Med..

[40] Yang L (2020). Study of cardiovascular disease prediction model based on random forest in eastern China. Sci. Rep..

[41] Su X (2020). Prediction for cardiovascular diseases based on laboratory data: An analysis of random forest model. J. Clin. Lab. Anal..

[42] Couronné R, Probst P, Boulesteix AL (2018). Random forest versus logistic regression: A large-scale benchmark experiment. BMC Bioinform..

[43] Ambler, G. & Benner, A. mfp: Multivariable fractional polynomials (2023).

[44] Zhang X (2020). Symptomatic intracranial hemorrhage after mechanical thrombectomy in Chinese ischemic stroke patients: The ASIAN score. Stroke.

[45] Greving JP (2014). Development of the PHASES score for prediction of risk of rupture of intracranial aneurysms: A pooled analysis of six prospective cohort studies. Lancet Neurol..

[46] DeLong ER, DeLong DM, Clarke-Pearson DL (1988). Comparing the areas under two or more correlated receiver operating characteristic curves: A nonparametric approach. Biometrics.

[47] Liaw A, Wiener MJRN (2002). Classification and regression by randomForest. R News.

[48] Weihs C, Ligges U, Luebke K, Raabe N, Baier D, Decker R, Schmidt-Thieme L (2005). klaR analyzing German business cycles. Data Analysis and Decision Support.

[49] Meyer, D. et al. e1071: misc functions of the department of statistics, probability theory group (formerly: E1071), TU Wien. **1** (2019).

[50] Hothorn T, Buehlmann P, Kneib T, Schmid M, Hofner B (2022). mboost: Model-based boosting. R Package Version.

