# Supplementary material for: Development and validation of an interpretable model for predicting sepsis mortality across care settings
Source: Sci Rep. 2024 Jun 13;14:13637. doi: 10.1038/s41598-024-64463-0 (PMC11176384; doi:10.1038/s41598-024-64463-0)
Supplement: Supplementary file 1 — Supplementary Tables. [file 41598_2024_64463_MOESM1_ESM.pdf]

Table S1. Initial vital signs and laboratory findings at time zero in the derivation dataset.

Variables	Total (N=7,436)	Survivor (N=4,688)	Non-survivor (N=2,748)
Initial vital signs			
Systolic blood pressure	96±29	97±29	95±29
Diastolic blood pressure	58±18	58±17	57±18
Heart rate	107±26	107±25	109±26
Respiratory rate	24±5	24±5	25±5
Body temperature	37.3±1.3	37.5±1.3	37.0±1.2
Initial laboratory findings			
WBC	12.9±12	12.6±10.3	13.3±14.4
Hb	10.5±2.5	10.7±2.5	10.2±2.6
Platelet	176±123	182±120	165±127
BUN	40±29	36±27	46±31
Creatinine	1.9±1.8	1.8±1.7	2.1±1.8
AST	129±559	92±274	192±843
ALT	68±217	59±190	82±256
Bilirubin	2.0±3.8	1.7±3.0	2.6±4.8
Albumin	2.9±0.7	3.0±0.7	2.7±0.7
INR	1.5±1.0	1.4±0.8	1.6±1.2
CRP	12.9±10.7	12.2±10.5	14.3±10.9
Lactate (mmol/L)	4.1±3.5	3.3±2.8	5.3±4.2

Abbreviations: WBC, white blood cell; Hb, hemoglobin; BUN, blood urea nitrogen; AST, aspartate aminotransferase; ALT, alanine aminotransferase; INR, international normalized ratio; CRP, C-reactive protein.

Data are presented as means ± standard deviations.

Table S2. Predictors of 28-day mortality after sepsis diagnosis using logistic regression analysis with the derivation dataset

Risk factors	Estimated Coefficient	Standard Error	Odds ratio	95% CI	P value
Age	0.016	0.002	1.02	1.011–1.021	,<0.001
Clinical frailty scale (1–3 points)			Reference		
4–5 points	0.540	0.089	1.72	1.442–2.041	<0.001
6–9 points	0.877	0.079	2.40	2.057–2.807	<0.001
Presence of malignancy	0.834	0.063	2.30	2.036–2.602	<0.001
SOFA score	0.105	0.010	1.11	1.088–1.133	<0.001
Sepsis caused by respiratory infection	0.688	0.061	1.99	1.765–2.242	<0.001
CRRT use	0.811	0.082	2.25	1.914–2.642	<0.001
Body temperature (>38°C, BT)			Reference		<0.001
36°C≤ BT ≤38°C	0.462	0.070	1.59	1.383–1.820	<0.001
<36°C	0.892	0.107	2.44	1.978–3.009	<0.001
Albumin (≥3.5 g/dL)			Reference		
2.5 g/dL≤albumin <3.5 g/dL	0.423	0.085	1.53	1.291–1.804	<0.001
<2.5 g/dL	0.864	0.097	2.37	1.963–2.867	<0.001

INR (>1.3)	0.326	0.062	1.39	1.227–1.563	<0.001
CRP (mg/dL)	0.010	0.003	1.01	1.005–1.016	<0.001
Lactic acid (≥4 mmol/L)	0.787	0.062	2.20	1.946–2.478	<0.001

Abbreviations: SOFA, Sequential Organ Failure Assessment; CRRT, continuous renal replacement therapy; BT, body temperature; INR, International normalized ratio; CRP, C-reactive protein.

Table S3. Evaluating Model Performance via Area Under the ROC Curve, Calibration Plots, Hosmer-Lemeshow Test, and Brier Score in the Derivation Dataset

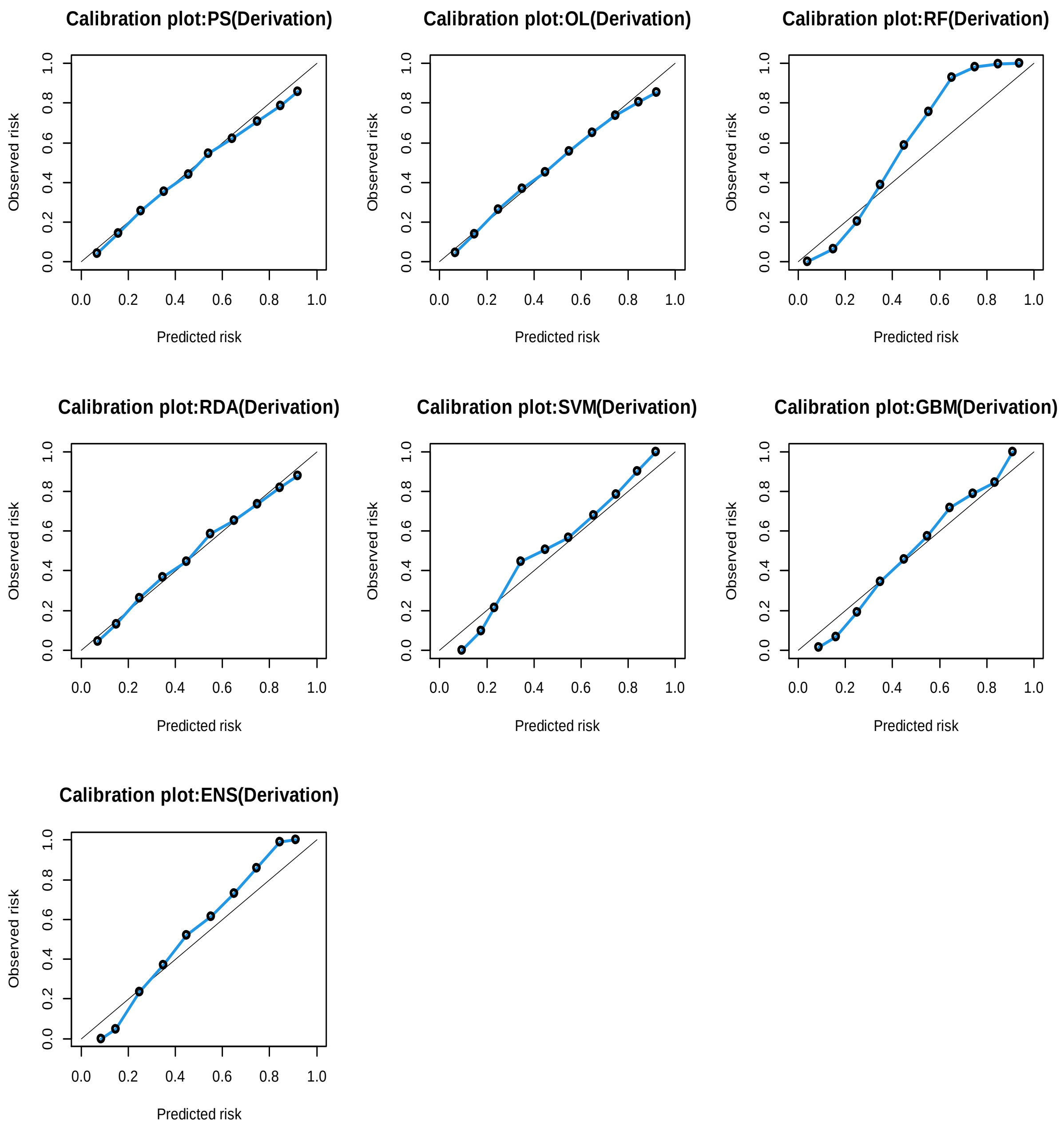

Method	AUC	95% confidence interval	Hosmer-Lemeshow test	Brier Score
PS	0.7743	0.763–0.785	28.8	0.184
OL	0.7787	0.768–0.790	15.2	0.182
RF	0.9632	0.959–0.967	613.7	0.090
RDA	0.7780	0.767–0.789	19.0	0.181
SVM	0.8196	0.809–0.830	122.5	0.168
GBM	0.7718	0.760–0.783	109.3	0.187
ENS	0.8545	0.846–0.863	268.8	0.155

Abbreviations: PS, point system; OL, ordinary logistic regression; RF, random forest; RDA, regularized discriminant analysis; SVM, support vector machine; GBM, gradient-boosting machine; ENS, ensemble method; AUC, area under the ROC curve.

Table S4. Evaluating Model Performance via Area Under the ROC Curve, Calibration Plots, Hosmer-Lemeshow Test, and Brier Score in the Cross Validation.

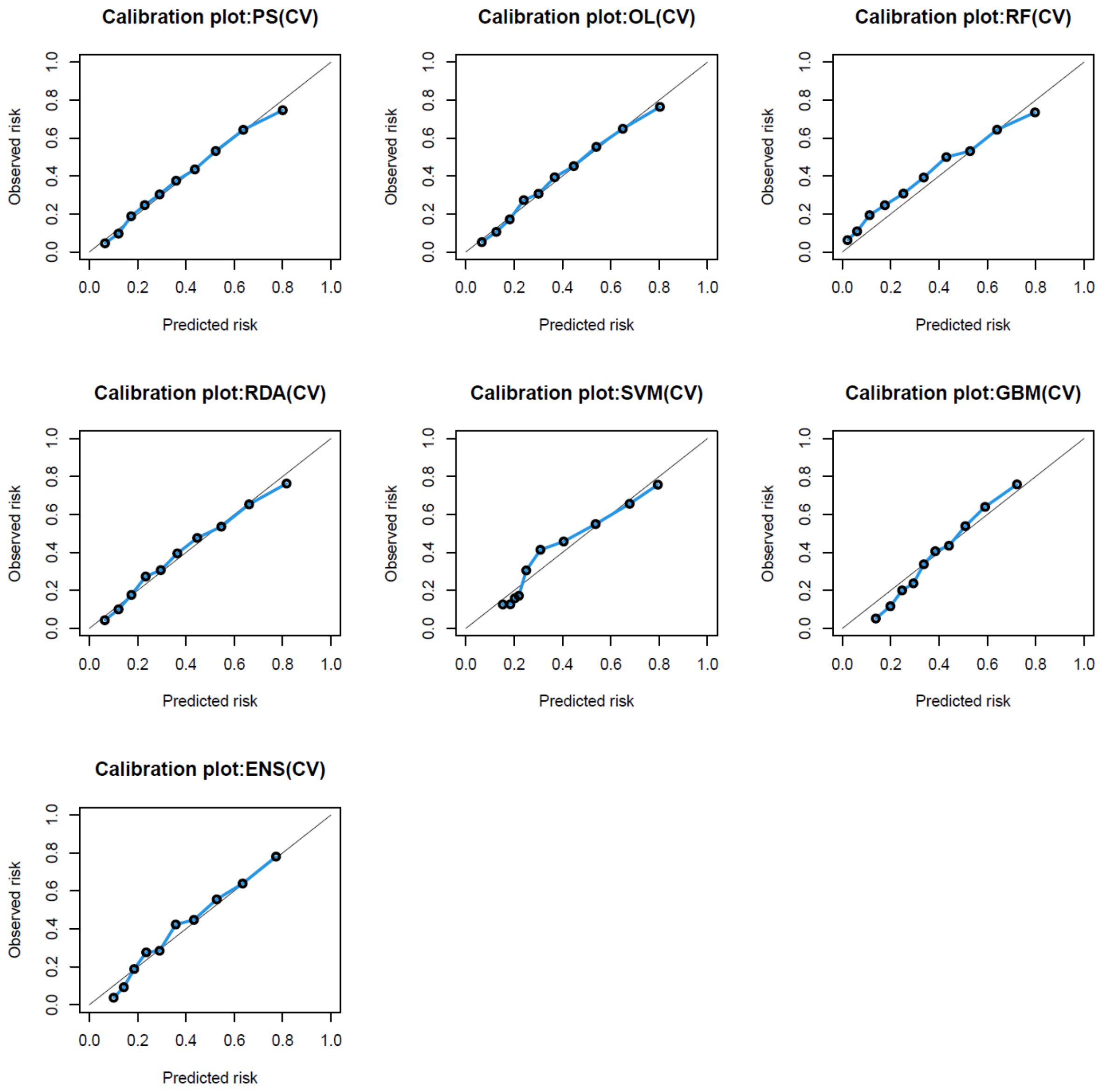

Method	AUC	95% confidence interval	Hosmer-Lemeshow test	Brier Score
PS	0.7722	0.747–0.797	11.74	0.185
OL	0.7765	0.752–0.802	12.89	0.184
RF	0.7693	0.744–0.795	50.24	0.189
RDA	0.7764	0.751–0.801	14.88	0.183
SVM	0.7650	0.739–0.791	1711.55	0.189
GBM	0.7695	0.744–0.795	31.13	0.188
ENS	0.7754	0.750–0.800	63.91	0.182

Abbreviations: PS, point system; OL, ordinary logistic regression; RF, random forest; RDA, regularized discriminant analysis; SVM, support vector machine; GBM, gradient-boosting machine; ENS, ensemble method; AUC, area under the ROC curve.

Table S5. Evaluating Model Performance via Area Under the ROC Curve, Calibration Plots, Hosmer-Lemeshow Test, and Brier Score in the Validation dataset.

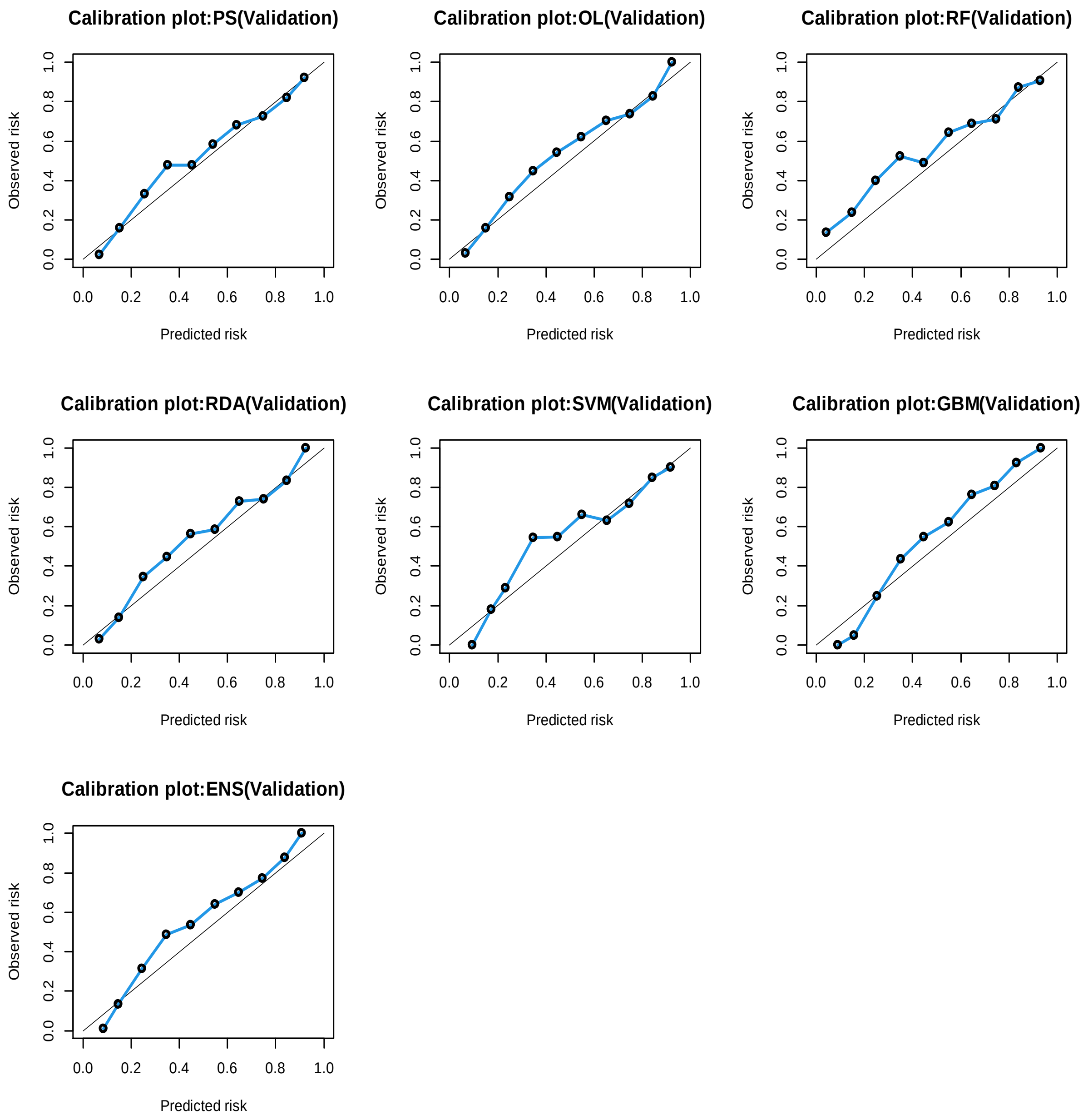

Method	AUC	95% confidence interval	Hosmer-Lemeshow test	Brier Score
PS	0.7717	0.752–0.792	41.7	0.195
OL	0.7790	0.759–0.799	42.8	0.193
RF	0.7652	0.745–0.786	192.9	0.204
RDA	0.7779	0.758–0.798	56.6	0.195
SVM	0.7536	0.732–0.775	67.8	0.206
GBM	0.7753	0.756–0.795	74.0	0.197
ENS	0.7807	0.761–0.800	63.3	0.195

Abbreviations: PS, point system; OL, ordinary logistic regression; RF, random forest; RDA, regularized discriminant analysis; SVM, support vector machine; GBM, gradient-boosting machine; ENS, ensemble method; AUC, area under the ROC curve.

Table S6. Mortality risk according to total point of new sepsis mortality model.

Point	Risk	Point	Risk	Point	Risk	Point	Risk
0	1.38	19	12.03	38	57.21	57	92.89
1	1.55	20	13.36	39	60.12	58	93.65
2	1.75	21	14.81	40	62.96	59	94.32
3	1.97	22	16.39	41	65.71	60	94.93
4	2.21	23	18.10	42	68.36	61	95.48
5	2.49	24	19.95	43	70.90	62	95.97
6	2.79	25	21.93	44	73.31	63	96.41
7	3.14	26	24.06	45	75.59	64	96.80
8	3.52	27	26.32	46	77.74	65	97.15
9	3.96	28	28.71	47	79.75	66	97.47
10	4.44	29	31.23	48	81.62	67	97.75
11	4.98	30	33.86	49	83.35	68	98.00
12	5.57	31	36.60	50	84.95	69	98.22
13	6.24	32	39.42	51	86.42	70	98.42
14	6.98	33	42.32	52	87.77	71	98.59
15	7.80	34	45.28	53	89.00	72	98.75
16	8.71	35	48.26	54	90.12	73	98.89
17	9.71	36	51.26	55	91.14		
18	10.82	37	54.25	56	92.06		

Table S7. IRB numbers in the derivation dataset (KSA 3)

Number	Institution	IRB number
1	Asan Medical Center	2018-0675
2	Chonnam National University Hospital	CNUH2029075
3	Chungnam National University Hospital	2019-11-048-001
4	Chungnam National University Sejong Hospital	2020-08-017
5	Daegu Catholic University Medical Center	CR-18-163-L
6	Hallym University Sacred Heart Hospital	2018-09-004
7	Hanyang University Guri Hospital	2020-08-015
8	Inje University Sanggye Paik Hospital	2018-08-014
9	Jeju National University Hospital	2018-06-012
10	Jeonbuk National University Hospital	CUH-2018-10-027
11	Kangwon National University Hospital	2018-08-004-001
12	Korea University Anam Hospital	2019AN0027
13	Pusan National University Yangsan Hospital	05-2019-092
14	Samsung Medical Center	2018-05-108
15	Seoul National University Bundang Hospital	B-1810-500-402
16	Seoul National University Hospital	H-1808-135-967
17	Severance Hospital	4-2020-0706
18	Ulsan University Hospital	UUH2018-08-003
19	Yeungnam University Medical Center	2020-08-051
20	Inje University Ilsan Paik Hospital	2021-08-003

Abbreviations**:** IRB, institutional review board; KSA, Korean Sepsis Alliance.

Table S8. IRB numbers in the validation dataset (KSA 4)

Number	Institution	IRB number
1	Seoul National University Bundang Hospital	B-1810-500-402
2	Samsung Medical Center	2018-05-108
3	Asan Medical Center	2018-0675
4	Inje University Sanggye Paik Hospital	2018-08-014
5	Seoul National University Hospital	H-1808-135-967
6	Hallym University Sacred Heart Hospital	2018-09-004
7	Hanyang University Guri Hospital	2020-08-015
8	Inje University Ilsan Paik Hospital	2021-08-003
9	Kangwon National University Hospital	2018-08-004-001
10	Chungnam National University Hospital	2019-11-048-001
11	Chungnam National University Sejong Hospital	2020-08-017
12	Jeonbuk National University Hospital	CUH-2018-10-027
13	Chonnam National University Hospital	CNUH2029075
14	Pusan National University Yangsan Hospital	05-2019-092
15	Jeju National University Hospital	2018-06-012

Abbreviations: IRB, institutional review board; KSA, Korean Sepsis Alliance.
